# ^225^Ac-PSMA-617 radioligand therapy of de novo metastatic hormone-sensitive prostate carcinoma (mHSPC): preliminary clinical findings

**DOI:** 10.1007/s00259-023-06165-9

**Published:** 2023-03-03

**Authors:** Mike Sathekge, Frank Bruchertseifer, Mariza Vorster, Ismaheel O. Lawal, Kgomotso Mokoala, Janet Reed, Letjie Maseremule, Honest Ndlovu, Khanyi Hlongwa, Alex Maes, Alfred Morgenstern, Christophe Van de Wiele

**Affiliations:** 1 Department of Nuclear Medicine, University of Pretoria & Steve Biko Academic Hospital, Pretoria, 0001 South Africa; 2 Nuclear Medicine Research Infrastructure (NuMeRI), Pretoria, South Africa; 3 European Commission, Joint Research Centre, Karlsruhe, Germany; 4 Department of Nuclear Medicine, University of Kwa-Zulu Natal & Inkosi Albert Luthuli Central Academic Hospital, Durban, South Africa; 5 Katholieke University Leuven, Kortrijk, Belgium; 6 Ghent University, Ghent, Belgium

**Keywords:** ^225^Ac-PSMA, mHSPC, Therapy response, PSA response, Prostate carcinoma

## Abstract

**Purpose:**

^225^Ac-PSMA-617 has demonstrated good anti-tumor effect as a treatment option for metastatic castration-resistant prostate cancer (mCRPC) patients. No study has previously assessed treatment outcome and survival following ^225^Ac-PSMA-617 treatment of de novo metastatic hormone-sensitive prostate carcinoma (mHSPC) patients. Based on the potential side effects that are known and explained to the patients by the oncologist, some of the patients refused the standard treatment and are seeking alternative therapies. Thus, we report our preliminary findings in a retrospective series of 21 mHSPC patients that refused standard treatment options and were treated with ^225^Ac-PSMA-617.

**Methods:**

We retrospectively reviewed patients with histologically confirmed de novo treatment-naïve bone ± visceral mHSPC that were treated with ^225^Ac-PSMA-617 radioligand therapy (RLT). Inclusion criteria included an Eastern Cooperative Oncology Group (ECOG) performance status of 0 to 2, treatment-naive bone ± visceral mHSPC, and patients refusal for ADT ± docetaxel, abiraterone acetate, or enzalutamide. We evaluated the response to treatment using prostate-specific antigen (PSA) response and the progression-free survival (PFS) and overall survival (OS) as well as the toxicities.

**Results:**

Twenty-one mHSPC patients were included in this preliminary work. Following treatment, twenty patients (95%) had any decline in PSA and eighteen patients (86%) presented with a PSA decline of ≥ 50% including 4 patients in whom PSA became undetectable. A lower percentage decrease in PSA following treatment was associated with increased mortality and shorter progression-free survival. Overall, administration of ^225^Ac-PSMA-617 was well tolerated. The commonest toxicity seen was grade I/II dry mouth observed in 94% of patients.

**Conclusions:**

Given these favorable results, randomized prospective multicenter trials assessing the clinical value of ^225^Ac-PSMA-617 as a therapeutic agent for mHSPC administered either as monotherapy or administered concomitant with ADT are of interest.

## Introduction

Prostate carcinoma is the most frequent malignancy and the second leading cause of cancer-related death in men [1].

At the time of diagnosis, the vast majority of newly diagnosed prostate carcinomas is either confined to the prostate or extends loco-regionally into surrounding structures or surrounding lymph nodes [2]. An estimated 5% of all prostate carcinoma patients, however, presents with de novo metastatic disease or metastatic hormone-sensitive prostate carcinoma (mHSPC) at the time of diagnosis [3]. According to current clinical guidelines based on randomized clinical trials, patients with high-volume mHSPC should be treated with standard androgen deprivation therapy (ADT) and abiraterone acetate (AA) with prednisone (P) or docetaxel (DOC) given adding either AA or DOC to ADT treatment was shown to significantly improve overall survival of mHSPC, as apalutamide and local RT for low-volume mHSPC [2, 4–6]. In the absence of a prospective randomized head-to-head comparison demonstrating superiority of one approach over the other, selection of either treatment option, ADT + AA+P versus ADT +DOC, is currently based taking into consideration disease burden, quality of life, duration of therapies, underlying comorbidities, wide respect to side effects, and patient preferences for treatment. While ADT + AA+P or ADT +DOC are usually well tolerated by fit and motivated patients, this is not the case in elderly, frail men presenting with important comorbidities. Accordingly, for the latter patients in whom mono-ADT is currently the treatment of choice, novel less toxic treatment options are warranted.

^225^Ac-PSMA-617 is a novel treatment option for prostate carcinoma patients and preliminary results obtained in patients with advanced prostate carcinoma have shown its therapeutic potential based on its high linear energy transfer (LET)-value related to its decay scheme, with 6 daughter products 221-Francium, 217-Astatine, 213-Bismuth, 213-Polonium, 209-Lead, and 209-Thallium (^221^Fr, ^217^At, ^213^Bi, ^213^Po, ^209^Pb and ^209^Tl) with several *α*- and *β*-decays [7–14]. In the studies reported to date using this agent, toxicities encountered using this agent are grade I or II xerostomia, observed in 85% of patients, anemia encountered in 37% of patients (predominantly grade I and II and no grade IV), and renal impairment encountered in 32% of patients (grade IV in 3% of patients only) [7–16]. Overall, side effects proved more prevalent among patients with a super-scan pattern.

Here, we report preliminary findings in a series of 21 bone ± visceral mHSPC patients (stage M1b or M1c) that refused standard treatment options and were treated with ^225^Ac-PSMA-617.

## Methods

This is a retrospective review of patients with histologically confirmed de novo treatment naïve bone ± visceral mHSPC that were treated with ^225^Ac-PSMA-617 radioligand therapy (RLT). Inclusion criteria included an Eastern Cooperative Oncology Group (ECOG) performance status of 0 to 2, treatment-naive bone ± visceral mHSPC, and patients refusal for ADT ± docetaxel, abiraterone acetate, or enzalutamide. Patients were aware that treatment of choice is widely available mono-ADT as well as the possibility of optional radiotherapy to the primary prostate tumor in low-volume patients [5, 6]. Unfortunately, except for ADT, the university hospital does not offer apalutamide, abiraterone acetate, or enzalutamide due to budgetary constraints. Exclusion criteria included a glomerular filtration rate below 60 mL/min, urinary tract obstruction as determined by ^99m^Tc-MAG renal scintigraphy, and bone marrow suppression. The decision to treat patients with ^225^Ac-PSMA-617 was made by the local interdisciplinary tumor board. Based on the potential side effects that are known and explained to the patients by the oncologist, some of the patients refused the standard treatment and are seeking alternative therapies. Patients were informed upfront of the fact that ^225^Ac-PSMA-617 treatment is not approved in South Africa as well as of the possible adverse events related to the treatment, respectively, xerostomia, bone marrow suppression, renal impairment, and potential currently unknown side effects. All patients gave written informed consent to undergo treatment with ^225^Ac-PSMA-617. The Human Research Ethics Committee of the Faculty of Health Sciences, University of Pretoria, approved this study (Ethics Reference Number: 173/2021).

### Patient preparation

Eligible patients had available baseline ^68^Ga-PSMA-11 PET/CT scan, clinical laboratory assessments which included urea and creatinine, electrolytes, liver function tests, and full blood count before ^225^Ac-PSMA-617 radioligand therapy. ^225^Ac-PSMA-617 treatments were administered every 8 weeks with activities being progressively decreased (de-escalation) from 8 MBq the first time to 6 or 4 MBq subsequently based on response to earlier administered treatments.

### Preparation and administration of ^225^Ac-PSMA-617

^225^Ac-PSMA-617 was radiolabelled as described previously. The initial administered activity was 8 MBq. For subsequent treatment cycles, administered activity was de-escalated to 7, 6, or 4 MBq based on response to earlier administered treatment. Treatments were repeated every 8 weeks. ^68^Ga-PSMA-11 PET/CT scan was repeated after each subsequent treatment cycle to determine the burden of residual tumor to guide dose de-escalation. The decision was based on response on the clinical, biochemical, and the on repeat ^68^Ga-PSMA-11 PET/CT as previously described [10]. This strategy is established on the principle of tumor sink effect in which more radioligand is available for binding in normal organs with reducing tumor bulk induced by successful treatment [17].

### Safety

Repeat clinical laboratory assessments which included urea and creatinine, electrolytes, liver function tests, full blood count, and glomerular filtration rate (GFR) were done at baseline, prior to each therapy cycle, 4 weeks after each cycle, and in 12-week intervals throughout follow-up. Severity of hematologic adverse events was graded based on Common Terminology Criteria for Adverse Events (CTCAE), version 5.0. Grade ≥ 3 toxicities were characterized as significant.

### Treatment response evaluation

PSA response was assessed according the Prostate Cancer Working Group 3 (PCWG3) criteria; that is, a PSA decline > 50% of the baseline was deemed clinically significant [18]. Follow-up ^68^Ga-PSMA-11 PET/CT (performed at baseline, prior to each treatment cycle, and every 12 weeks following treatment completion until disease progression) was used to define resolution of initially identified metastatic lesions on the baseline PET/CT scan.

### Statistical analysis

Statistical analysis was performed using SPSS, version 28.0 (IBM SPSS). Based on the results of the Kolmogorov-Smirnov test, the appropriate tests were used to compare means (paired Student *t* test and ANOVA if normally distributed and Mann-Whitney test and Kruskal-Wallis test if not normally distributed). Univariate and regression analysis was performed on data dichotomized according the median value for continuous variables. For quantitative continuous variables, the median was used to obtain two approximately equal-sized groups for logrank testing (10 versus 11 pts). For other variables such as ECOG-score and Gleason score, the cut-off yielding the best equilibrated group sizes was used. For radiological response assessment, patients were dichotomized as follows, CR +PR versus SD + progression. In other cases (negative PSMA PET, PSA decrease > 50%, and M1b versus M1c), it is self-explanatory how two groups for comparison were obtained. The predictive value of dichotomized variables and other clinical risk factors for disease control and OS were estimated by the Kaplan-Meier method and logrank testing. Multivariate analysis was performed using Cox-regression. Finally, the Chi-square test was used to determine differences in proportion when appropriate.

## Results

### Patient characteristics

Patient characteristics are shown in Table 1. Twenty-one treatment-naive HSPC patients, respectively, 15 M1b and 6 M1c patients, were included in the study; median age was 67 years (range 53–80 years). ECOG scores were as follows, 0 in 8 patients, 1 in 9 patients, 2 in 3 patients, and 3 in 1 patient. Fifteen patients suffered from stage M1b disease and 6 patients from stage M1c disease (in the lungs in 2 patients, in lungs and liver in 1 patient, in the liver in 1 patient, in liver and brain in 1 patient, and finally in the brain in 1 patient). PSA, Hb, and alkaline phosphatase levels, platelet and WBC counts pre-treatment are shown in Table 1.

**Table 1:** Patient characteristics

No. of patients included	21
Median age (yrs)	67
ECOG score of 0 or 1	17
ECG score of > 2	4
Gleason score ISUP grade (range)	4 (1–5)
Median baseline PSA level (range) (ng/mL)	196.7 (4.58–5000.00)
Median alkaline phosphatase levels (range) (IU/L)	313 (69–2148)
Median hemoglobin level (range) (g/dL)	10.4 (6.9–15.7)
Median white blood cell count (range) (/μL)	6.1 (3.82–10.72)
Median platelet count (range) (/μL)	299,000 (157,000–687,000)

**Fig. 1:**
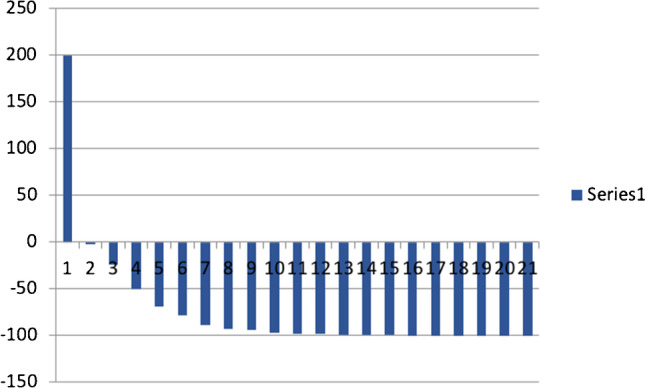
Waterfall plot demonstrating percentage change in PSA levels after treatment with ^225^Ac-PSMA-617 in studied patients (*x*-axis= number of patients, *y*-axis= percentage change)

**Fig. 2:**
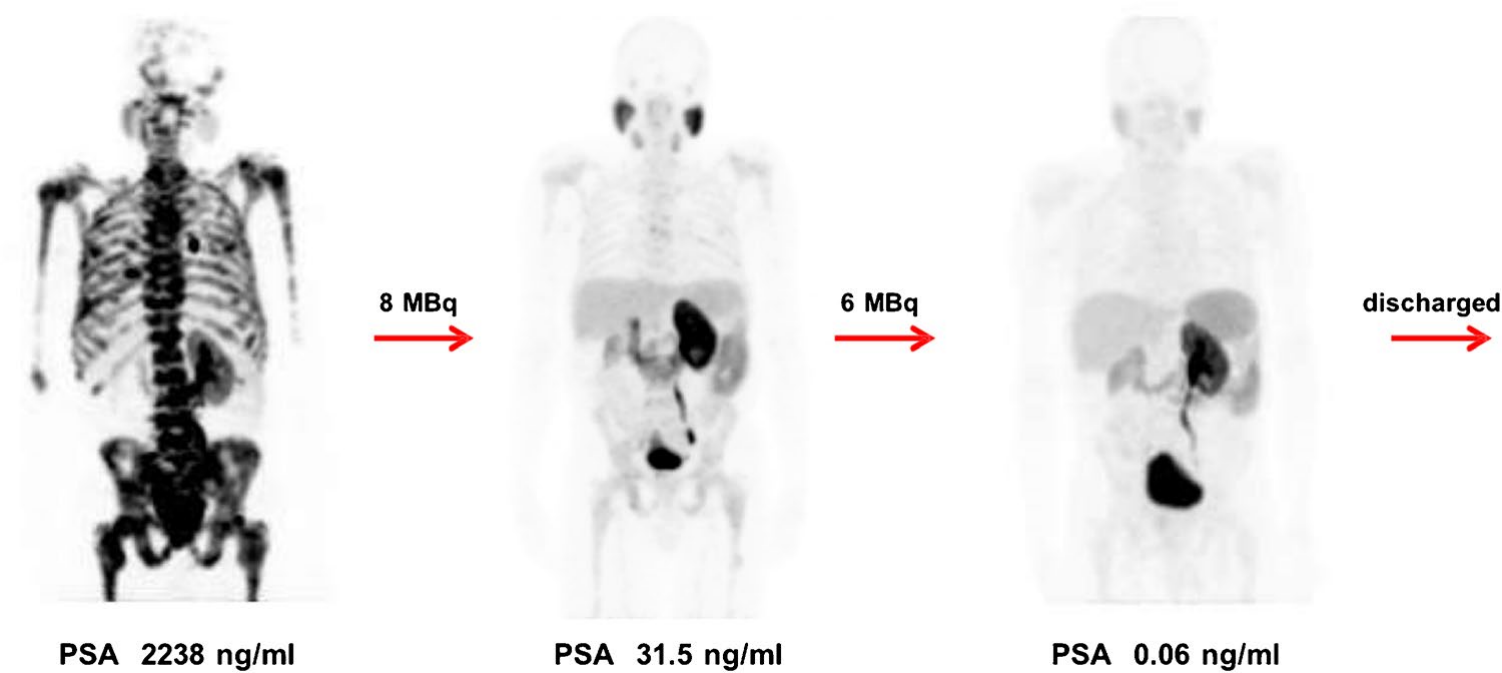
A good responder treatment-naïve patient who presented with ^68^Ga-PSMA-11 (SUV max =22.57) avid extensive bone metastasis at primary diagnosis was discharged after two cycles of ^225^Ac-PSMA-617 with de-escalating activities of 8/6 MBq. His follow-up ^68^Ga-PSMA-11 PET/CT scan was negative PFS and his OS was 22 months

**Fig. 3:**
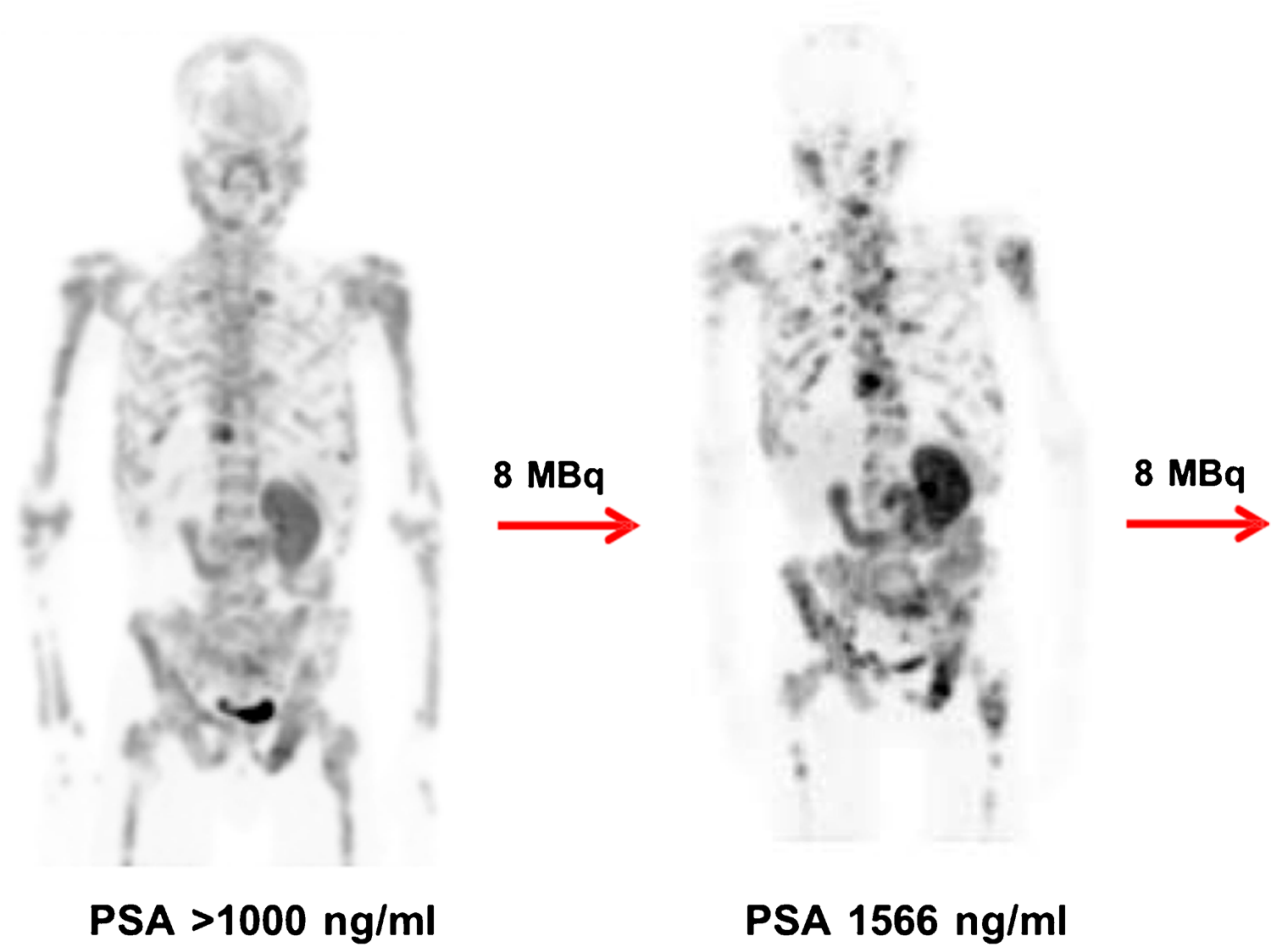
A poor responder treatment-naïve patient who presented with some ^68^Ga-PSMA-11 uptake (SUV max =10.25) extensive bone metastasis at primary diagnosis was discharged after two cycles of ^225^Ac-PSMA-617 with no de-escalating activities of 8/8 MBq. His follow-up ^68^Ga-PSMA-11 PET/CT scan was more avid than the baseline and his OS was 5 months

A total number of 68 cycles were administered (median of 3, range 2–6). Six patients received 2 cycles, 8 patients received 3 cycles, 4 patients received 4 cycles, 2 patients received 5 cycles, and 1 patient received 6 cycles. Eighteen patients (86%) presented with a PSA drop of greater than or equal to 50% and 20 patients (95%) had any drop in PSA (see Fig. 1). PSA became undetectable in 4 patients. ^68^Ga-PSMA-PET images became negative in 7 patients; that is, avidity was similar to background bloodpool activity. The response assessment decision was based on the clinical, biochemical, and the on repeat ^68^Ga-PSMA-11 PET/CT as previously described [10], leading to dose de-escalation in the case of good response (see Fig. 2) or maintaining the same dose if the response is not good (see Fig. 3). Although the study numbers are small, these examples seem to support that PSMA tumor expression on PET images appears as one of the predictors of the outcome as suggested by some of the recently published work [19–22]. All patients had bone metastases and the number was too small to differentiate between various visceral metastases. Hence, the results, OS, did not prove significantly different between M1b and M1c disease (log-rank test, *p*= 0.478).

### Safety

Administration of ^225^Ac-PSMA-617 was well tolerated. The commonest toxicity seen was grade I/II dry mouth observed in 94% of patients. There was no reversibility seen on completion; patients tend to adjust to the new lifestyle of living with xerostomia by using over-the-counter saliva substitutes (moisturizing mouth spray) and drinking water frequently. No dry eye was observed in this retrospective analysis. At the group level, hemoglobin levels as well as platelet and white blood cell did not change significantly post-treatment, respectively, *p*= 0.945, *p*=0.598, and *p*=0.841. At the individual level, none of the patients presented with a significant change in platelet or white blood cell count post-treatment when compared to pre-treatment values. The mean pre- and post-hemoglobin level was 10.4 (range, 6.9–15.7 g/dL) versus 9.5 g/dL (range, 5.3–14.2 g/dL); the mean pre- and post-platelet count was 299.5,000 (range, 157,000–687,000/μL) versus 252.5,000/μL (range, 132,000–404,000/μL); the mean pre- and post-white blood cell count was 6.1 (range, 3.82–10.72/μL) versus 6.1/μL (range, 3.69–11.37/μL). However, in ten patients, a significant decrease in Hb level post-treatment was observed. Given the wide range of pre-treatment Hb levels, toxicity assessment according to standard criteria was not deemed meaningful. Thus, we assessed the percentage decrease in HB-levels as compared to baseline values in this patient group. Median decrease in HB-level as compared to baseline values was 25% (range 13–72%).

### Overall survival

**Fig. 4:**
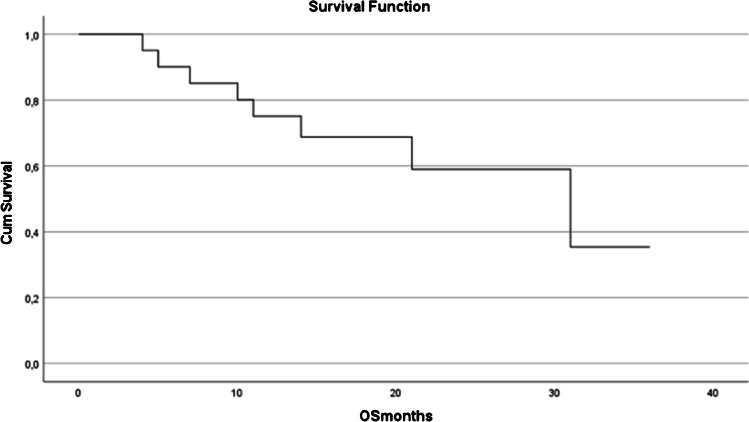
Overall survival curve of the cohort studied

Overall survival of the whole patient population is demonstrated in Fig. 4. Estimated median overall survival for the entire patient population was 31 months (CI 12.8–49.2 months). In univariate analysis, both the age (relatively younger patients lived more) (*p*= 0.045) and PSA decline (PSA decline > 50%) dichotomized according the median proved to be significantly associated with a favorable OS (*p*= 0.001) (see Table 2). In multivariate analysis, only PSA decline proved statistical relevant (*p*=0.003). Median estimated OS for patients with a PSA decline smaller than the median was 11 months (CI 8.1–13.8 months) whereas the median OS of those patients with a PSA decline > the median PSA decline was not yet reached at the date of latest follow-up (36 months).

**Table 2:** Univariate analysis of the relationship between studied variables and survival

Variable	OS	PFS
Age	**0.045**	0.122
ECOG score	0.442	0.333
Gleason score	0.827	0.158
Baseline PSA	0.599	0.613
PSA median	**0.001**	**0.015**
PSA undetectable	0.521	0.235
Nb of treatment cycles	0.151	0.204
Alkaline phosphatase	0.660	0.784
Hemoglobin	0.223	0.219
Platelets	0.731	0.317
White blood cell count	0.076	0.166
Radiological response	0.110	0.075
Negative PSMA PET	0.110	0.075
Bone ± visceral metastases	0.478	0.841

Significant *p*-values are highlighted in bold

Gleason score was not a significant variable

### Progression-free survival

PSA decline proved the only significantly variable in univariate (*p*=0.013) as well as in multivariate analysis. PFS for patients with a PSA decline > the median was not yet reached at the time of latest follow-up whereas that for patients with a PSA decline < the median was 9.0 months (CI 1.8–16.2 months).

## Discussion

A prospective pilot study with ^177^Lu-PSMA appeared to be a feasible and safe treatment modality in ten patients with low-volume mHSPC in seeking patient alternative therapies. Similar studies have never been done with ^225^Ac-PSMA [23]. Hence, our retrospective study is the first one to evaluate the preliminary clinical experience using ^225^Ac-PSMA. In line with previous reports on ^225^Ac-PSMA-617 treatment, in the series presented, 86% of mHSPC patients (18/21) had a ≥ 50% PSA reduction of their initial PSA-value following ^225^Ac-PSMA-617 treatment and both baseline PSA values and alkaline phosphatase levels proved unrelated to the percentage PSA reduction confirming the high level of radiobiological effectiveness of ^225^Ac, related to its 100keV/micron LET, obviating the need of cellular oxygenation for achieving cell death [24, 25].

In the series presented, both the degree of PSA reduction and advanced age at the time of diagnosis proved a significant predictor for overall survival in univariate analysis. An advanced age at diagnosis has been previously linked to a higher likelihood of presenting with de novo metastatic prostate carcinoma and worse prostate cancer-specific survival [26, 27]. In a study by Scosyrev et al. on data from 464,918 patients diagnosed with prostate carcinoma between 1998 and 2007, the authors found that when compared to younger patients (aged < 75 years), older patients were more likely to present with advanced disease and have a greater risk of death from prostate carcinoma despite higher death rates from competing causes and to contribute to more than half of all prostate carcinoma related deaths [28]. Potential causes for more aggressive disease in elderly patients reported in literature include less PSA screening and higher rates of hypogonadism in older men leading to the emergence of cancer less driven by androgens with a more castrate-resistant profile [26, 29, 30]. Low serum levels of testosterone as found in older men have also been previously associated with a higher Gleason score on prostatectomy biopsies [31, 32]. In the series presented, age proved unrelated to the Gleason score of the primary tumor as well as to the baseline PSA levels, reflecting disease extent. However, response to treatment as assessed by the percentage reduction in PSA did prove significantly lower at a higher age and unrelated to the number of treatment cycles administered in favor of a more aggressive character of the underlying prostate carcinoma in these patients.

In multivariate analysis only, the reduction in PSA levels following treatment administration proved predictive for OS as well as the only predictive factor in univariate and multivariate analysis for progression free survival. Given that only 3 patients demonstrated a PSA decline inferior to 50% (cut-off recommended by the PCWG3 group), we used the median of the percentage change in PSA, respectively −98%, to create two equal-sized groups for statistical comparison using the Kaplan-Meier analysis and the log-rank test as customary when applying this test. Using this cut-off, median estimated OS and PFS were respectively 11 months (CI 8.1–13.8 months) and 9.0 months (CI 1.8–16.2 months) for the group with a response inferior to à 98% reduction, whereas for the group that showed a reduction superior or equal to a 98% decrease in PSA levels, median OS and PFS were not yet reached at the time of latest follow-up, respectively 36 months.

Results obtained in this series are difficult to compare directly to those studies that have defined the current standard of care for treatment of de novo mHSPC given the absence of a direct comparison to the current standard of care in our study on the one hand and the specific inclusion of M1b (± M1c) patients included in this trial on the other hand. With the exception of the LATITUDE trial which compared ADT + placebo to ADT + abiraterone acetate (AA) and prednisone (P) in de novo M1b and or M1c prostate carcinoma, all other studies either comparing ADT to ADT +docetaxel (STAMPEDE, CETUG, and CHAARTED trial), ADT to ADT+AA+P (STAMPEDE arm G trial), ADT ± docetaxel to ADT + enzalutimide ± docetexal (ENZAMET trial), and ADT to ADT + apalutamide (TITAN trial) included a mix of patients with the range of newly diagnosed stage M1 prostate carcinoma patients included varying from 48 (STAMPEDE trial) to 78% (TITAN trial) [33–39]. In the LATITUDE trial, including a similar subset of patients as the ones included in our study, 3-year OS was 66% for the arm receiving ADT+AA+P versus 49% in the control arm (ADT + placebo).

When considering the entire patient cohort in our study, approximately 50% of the patients were still alive at 32 months follow-up (see Fig. 4) suggesting that ^225^Ac-PSMA-617 treatment may be more or less as efficient when compared to ADT alone but less efficient when compared to ADT+AA+P. However, our study included only a small number of patients and results of randomized clinical trials, including well-selected patients, do not necessarily translate in real-world survival improvement of de novo metastatic prostate carcinoma patients. In this regard, Helgstrand et al. analyzed the incidence and mortality data of patients with de novo metastatic prostate carcinoma included in the SEER database and in the Danish Prostate Cancer Registry. In patients diagnosed between 2000 and 2009, the median OS was 22 months in SEER and 30 months in the Danish registry [40]. More recently, Cattrini et al. analyzed survival data from more than 26,000 patients included in the SEER database. These authors found a survival gain of only 4 months between patients diagnosed in 2011–2014 versus those diagnosed in 2000–2003 and 2004–2010; docetaxel which was approved by the FDA for treatment of mCRPC in 2004, and ARSi which was approved from 2011 onwards [41]. Thus, it appears that overall, our results obtained are in line with those derived from real-world survival analysis. Factors potentially contributing to the discrepant results between real-world results and those derived from randomized trials include patients’ ineligibility or refusal of anticancer treatments (as was the case in the series presented), insurance issues, intrinsic disease aggressiveness, or prior unavailability of drugs in a hormone-sensitive setting (enzalutamide and abiraterone acetate are not readily reimbursed in low-mid–income countries).

In terms to of the safety profile, salivary gland toxicity was noted in majority of our patients (94%), which is slightly more than we reported previously [9, 10, 14], and thus a call for concern to find the solution for xerostomia especially if this treatment is to be considered in the de novo mHSPC cohort. Xerostomia remains a challenge and limiting factor for ^2^^25^Ac-PSMA-; hence, some approaches continue to be investigated, excluding the suggested dose deescalating scheme from our group. Several workers have investigated the concept of co-administering lower activities ^225^Ac-PSMA-617 (mean 5.3 MBq) and ^177^Lu-PSMA-617 (mean 6.9 GBq) in mCRPC patients and found no grade 3 xerostomia and no treatment discontinuation was observed [42–45]. Another strategy is based on the fact that there might be a severe duct stenosis or obstruction; the study from Rathke et al. have showed a response to sialendoscopic duct dilatation and saline irrigation [46]. Other approaches that have been implemented in some clinics include external cooling [47, 48], orally administered monosodium glutamate [49, 50], and high-dose botulinum toxin injections [51], with preliminary encouraging data on the effectiveness. The hematologic and renal profiles of patients remained safe for the brief period of follow-up as none of the patients demonstrated grade 3/4 hematotoxicities or renal toxicities, similar to our recent report [15].

Unfortunately, there are several limitations to this study. It is a retrospective investigation with a small sample size and related statistical limitations. Additionally, no dosimetry has been performed. Furthermore, a limited worldwide production of ^225^Ac in general may limit its use in research and subsequently in the clinical practice.

Available data suggest that the incidence of de novo mHSPC is likely to increase in the course of the following years related to the recent trend towards less PSA screening. Because of their higher age and related comorbidities, de novo mHSPC can in some selected cases not receive chemotherapy or do not tolerate ADT± AA well. Accordingly, there is a need for more tolerable and efficacious treatment options of these patients. As suggested by our preliminary findings, ^225^Ac-PSMA-617 may just be such a treatment. Studies in larger patient populations confirming our results as well as multicenter randomized trials comparing patient outcome following ^225^Ac-PSMA-617 treatment to that obtained using standard of care treatment in mHSPC are mandatory.

### Availability of data and material

All data collected during the conduct of this study are included in this report.

## Declarations

**Ethics approval**

This study was performed per the ethical standard of our institutions and with the 1964 Helsinki Declaration and its later amendments or comparable ethical standards.
